# Whole-Genome Sequence of a Plant Growth-Promoting Strain, Serratia marcescens BTL07, Isolated from the Rhizoplane of Capsicum annuum L.

**DOI:** 10.1128/MRA.01484-19

**Published:** 2020-04-30

**Authors:** Sudipta Dutta, Amena Khatun, Dipali Rani Gupta, Musrat Zahan Surovy, M. Mahbubur Rahman, Nur Uddin Mahmud, Richard D. Emes, Andrew Warry, Helen M. West, Michèle L. Clarke, M. Nazmul Hoque, Muhammad Maqsud Hossain, Mohammad Abdus Salam, M. Tofazzal Islam

**Affiliations:** a Institute of Biotechnology and Genetic Engineering (IBGE), Bangabandhu Sheikh Mujibur Rahman Agricultural University (BSMRAU), Gazipur, Bangladesh; b School of Veterinary Medicine and Science, University of Nottingham, Nottingham, United Kingdom; c Advanced Data Analysis Centre, University of Nottingham, Nottingham, United Kingdom; d School of Bioscience, University of Nottingham, Nottingham, United Kingdom; e School of Geography, University of Nottingham, Nottingham, United Kingdom; f Faculty of Veterinary Medicine & Animal Science, BSMRAU, Gazipur, Bangladesh; g NSU Genome Research Institute (NGRI), North South University, Dhaka, Bangladesh; h Department of Genetics & Fish Breeding, Faculty of Fisheries, BSMRAU, Gazipur, Bangladesh; University of Maryland School of Medicine

## Abstract

Serratia marcescens strain BTL07, which has the ability to promote growth and suppress plant diseases, was isolated from the rhizoplane of a chili plant. The draft genome sequence data of the strain will contribute to advancing our understanding of the molecular mechanisms underlying plant growth promotion and tolerance to different stresses.

## ANNOUNCEMENT

Serratia marcescens is a Gram-negative plant growth-promoting bacterium isolated from various plant species (1, 2). S. marcescens strain BTL07 was isolated from the rhizoplane of a chili (Capsicum annuum L.) plant from BSMR Agricultural University Farm in Gazipur, Bangladesh, in 2011. In order to isolate epiphytic bacteria, chili root samples were washed thoroughly with sterilized distilled water and subsequently homogenized through vortexing for 1 min in 20 ml distilled water in a sterile test tube, and a serial dilution was made up to 1 × 10^−9^. Exactly 100-μl aliquots of each sample (1 × 10^−9^ dilution series) were spread on nutrient agar plates and incubated at 25°C for 48 h. Finally, morphologically distinct (color, size, and shape) single colonies were purified by repeated streak culture on the same medium. BTL07 was found to be positive for oxidase and catalase, production of indole-3-acetic acid (IAA), and growth in sodium chloride (NaCl) and caused lysis of zoospores of a notorious phytopathogenic oomycete, Phytophthora capsici (3). A bioassay revealed that the isolate stimulated cucumber seed germination and enhanced the growth of seedlings (3). The genomic DNA of S. marcescens strain BTL07 was extracted using the phenol-chloroform DNA extraction method (4) and was precipitated using isopropanol. Treatment with DNase-free RNase (Sigma Chemical Co., St. Louis, MO, USA) resulted in a final concentration of 0.2 μg DNA/ml at 37°C. Later, second-phase extraction and isopropanol precipitation were performed similarly. The DNA pellet was resuspended in Tris-EDTA (TE) buffer (10 mM Tris-HCl and 1 mM EDTA) at pH 8.0. Ultimately, the DNA pellet was stored at −20°C until sequencing. Whole-genome sequencing was carried out to discover the specific genes involved in different plant growth promotion mechanisms. The genomic DNA was used to construct a whole-genome sequencing library. We fragmented the samples with Covaris to around 550 to 600 bp. We then used the NEBNext Ultra DNA library prep kit for Illumina from New England BioLabs (catalog number E7370S) with 6 PCR cycles (https://international.neb.com/products/e7370-nebnext-ultra-dna-library-prep-kit-for-illumina#Product%20Information). The constructed libraries were sequenced using an Illumina MiSeq platform with 250-bp paired-end reads and 140× genome coverage. All software mentioned was used with default parameters unless otherwise specified. The genome sequencing generated 4,464,167 reads. The quality of the raw sequence data was initially checked using FastQC (http://www.bioinformatics.babraham.ac.uk/projects/fastqc/). The raw data were trimmed for quality and adapter contamination with Sickle version 0.991 (https://github.com/najoshi/sickle) and Scythe version 1.33 (https://github.com/vsbuffalo/scythe), respectively, and then assembled with SPAdes version 3.13 (5) using k-mer sizes (-k) set to 21, 33, 55, 77, 99, and 127 and the careful pipeline option. The sequence length of the draft genome was 5,270,445 bp, with 18 contigs and a genome coverage of 140×. The overall G+C content and *N*_50_ value were determined to be 59.5% and 1,537,585 bp, respectively, and the largest contig assembled was 1,589,761 bp.

Annotation was carried out using the NCBI Prokaryotic Genome Annotation Pipeline (PGAP), predicting 4,967 protein coding genes, 13 rRNAs, and 83 tRNAs (6). The predicted 16S rRNA gene sequence showed 100% identity to that of Serratia marcescens subsp. *marcescens* strain Db11 (GenBank accession number NZ_HG326223) and 99% to that of S. marcescens strain B3R3 (NZ_CP013046). Gene function was taken from the PGAP annotation supplemented with the RAST server (7). Metabolic cluster and metabolic model finding were performed by antiSMASH (8). A BLAST search was carried out to find the homologous genes (9).

Genome analyses divulged a number of orthologs to innate plant growth-promoting genes for facilitating sustained production, such as those encoding stimulation of seedling germination (*amyAS*) and growth promotion (*nasD*, *glnA*, and *nirD*; *phoABPR*, *acpho*, *phnCDEFGHJKLMNP*, *pqqCDEF*, and *gcd*; and *kdpABCDE* and *trkADGH*) by inducing NPK uptake (10–14), heat shock proteins (*dnaJK*, *groEL*, and *clpP*), cold shock proteins (*cspACDE* and *csh*), and trehalose biosynthesis (*otsAB*), involved in abiotic stress tolerance (10). Furthermore, some genes involved in biocontrol functions, for instance, production of hydrogen cyanide (*hcnABC*), cell wall-degrading enzyme (*chiDE*), and acetoin (*alsS* and *gdh*) (10, 11, 15, 16), and heavy metal efflux-mediated proteins (*arsB, czcD*, and *nikAB*) for arsenic, cadmium, cobalt, zinc, and nickel were incorporated in the genome of BTL07. Functional annotation also detected genes related to IAA phytohormones (*dhaS*, *bioA*, *patB*, and *aldA*), a siderophore (*entABCDES*), and antioxidant enzymes (*katEG*, *sodABC*, and *bsaA*) in this genome; these genes are likely to be linked with plant growth promotion by both direct and indirect pathways (10, 11).

### Data availability.

This whole-genome shotgun project has been deposited at GenBank under the assembly accession number GCA_009746745. The raw sequencing data have been deposited under BioProject accession number PRJNA590998.
